# Prevalence and nature of bullying in schoolchildren aged 10–14 years and its association with malocclusion: A cross-sectional study in the South East of the UK

**DOI:** 10.1177/14653125241235677

**Published:** 2024-03-04

**Authors:** Andrew DiBiase, Zaffie Cox, Michaela Rea, Lazaros Gonidis, Lindsey Cameron, Adam Rutland

**Affiliations:** 1 Maxillofacial Department, William Harvey Hospital, East Kent Hospitals University NHS Foundation Trust, Ashford, Kent, UK; 2 School of Psychology, University of Kent, Canterbury, Kent, UK; 3 Department of Psychology, Goldsmiths, University of London, London, UK; 4 Psychology, University of Exeter, Exeter, UK

**Keywords:** malocclusion, bullying, schoolchildren, frequency

## Abstract

**Objective:**

To investigate the prevalence of, and relationship between, bullying and malocclusion in schoolchildren aged 10–14 years in the South East of the UK.

**Design:**

Cross-sectional cohort study.

**Setting:**

Sixteen primary and secondary schools in South East of the UK.

**Participants:**

Schoolchildren aged 10–14 years who were consented to participate.

**Methods:**

The prevalence and nature of bullying were measured using a questionnaire. Traits of malocclusion and the need for orthodontic treatment were assessed by clinical examination and determined by the Index of Orthodontic Treatment Need (IOTN) Dental Health (DHC) and Aesthetic components (AC).

**Results:**

Complete data were collected for 698 participants. The number defined as being bullied was 68 (9.7%). There was no difference in the prevalence of bullying between gender, ethnicity or age. Higher rates of bullying were reported in mixed sex schools (*P* = 0.03). Participants with an increased overjet (*P* = 0.02) and/or a greater need for treatment, as measured by IOTN DHC (*P* = 0.01) and AC (*P* = 0.01), reported higher rates of bullying. While there was no difference in the overall prevalence of bullying between genders, boys were more likely than girls to experience physical bullying (*P* <0.001) and being called names (*P* = 0.03)

**Conclusion:**

A significant relationship was evident between being bullied and certain traits of malocclusion.

## Introduction

Bullying among schoolchildren is endemic within most education systems globally, with almost one in three students having been bullied by their peers at school at least once in the last month (UNESCO, 2019). Bullying can be defined as a practice of aggressive behaviour or intentional harm to which an individual is repeatedly exposed in a relationship characterised by an imbalance of power (Olweus, 1994). This can take the form of direct physical or verbal abuse, or indirect bullying via social exclusion, spreading of rumours or gossip. In recent years, there has been a significant increase in cyberbullying (Englander et al., 2017).

The Office for National Statistics in the UK found in 2017–2018 that 17% of adolescents aged 10–15 years reported being bullied in the last 12 months, with the proportion being higher in the younger age groups: 22% of 10-year-olds surveyed compared to 8% of the 15-year-olds (DfE, 2018). Of those who reported being bullied, 60% had experienced physical bullying while 89% said they had suffered some form of verbal bullying. Boys are more likely to experience physical bullying, while indirect forms of bullying, such as social isolation and rumour-spreading, is more common in girls (DfE, 2018).

Bullying can have both short- and long-term effects on mental and physical health. High levels of anxiety and depression have been found in young bullying victims, who report lower self-esteem and greater social isolation, lack a consistent friendship group and do poorly academically (Moore et al., 2017; Yuchang et al., 2019). Bullying is one of the major reasons for school absence (Brown et al., 2011). Bullying also leads to higher rates of depression and suicidality (Brunstein Klomek et al., 2007). In the long-term, adult victims of childhood bullying report higher levels of anxiety, depression, post-traumatic stress disorder, social isolation and poor physical health (Arseneault, 2018; Copeland et al., 2013; Gladstone et al., 2006; Sigurdson et al., 2014; Stapinski et al., 2014).

Physical appearance is one of the most frequently cited reasons for bullying in childhood. In a UK survey of 12–20-year-olds, of the 25% who reported being bullied, 47% reported this was due to their appearance (Ditch the Label, 2020). This can be directed at weight, height, body shape and physical attractiveness (Haegele et al., 2021; Ievers-Landis et al., 2019; Koyanagi et al., 2020; Puhl et al., 2013; Rosen et al., 2011; Voss and Mulligan, 2000; Wang et al., 2018).

It has been recognised for some time that aesthetically handicapping features of malocclusion may make a child or adolescent more susceptible to victimisation (Shaw et al., 1980). The reported prevalence of bullying about dentofacial features ranges from 7% (Shaw et al., 1980) to 47.8% (Baram et al., 2019), while bullying among children with malocclusion seeking treatment has been found to range from 13% (Seehra et al., 2011) to 38% (Fleming et al., 2008). An increase in the incidence of bullying has been linked to certain traits of malocclusion, such as an increased overjet and a more severe malocclusion (Seehra et al., 2011: Tristao et al., 2020). Being teased or bullied is cited as a motivating factor to seek orthodontic treatment (Bauss and Vassis, 2023; Fleming et al., 2008). However, the evidence to support this relationship to date is limited as it either looks at orthodontic patient groups, who will present with a disproportionately higher rate and severity of malocclusion, or in broader population groups, often relying on self-reported traits of malocclusion (Tristao et al., 2020). There is also variation in how bullying and malocclusion have been measured and classified, resulting in large heterogeneity between studies (Miranda e Paulo et al., 2022). The aim of this exploratory study was to address some of these limitations and extend our understanding of the relationship between bullying and malocclusion in young people.

The objectives of this initial exploratory study were to use both a questionnaire and clinical examination to investigate the prevalence of self-reported bullying in a group of schoolchildren aged 10–14 years in the UK, to investigate patterns of self-reported bullying experienced and how this varied with age, gender and type of school, and to investigate the extent to which self-reported bullying and type were associated with different traits of malocclusion.

## Methodology

This was a cross-sectional mixed-methods study, involving participant questionnaires and clinical examination, investigating the relationship between being bullied and the presence of one or more features of malocclusion in schoolchildren aged 10–14 years in the UK. Ethical approval was obtained from the London-Surrey Research Ethics Committee (no. 17/LO/0791)

### Participants and data collection

Participants were recruited from primary and secondary schools in London and the South East of the UK. Students aged 10–14 years were recruited, as previous research suggests bullying is more prevalent in this age range (DfE, 2018). This is also the age when most orthodontic treatment is carried out.

Schools were approached based on location for practicality of data collection and demographics with the aim of including students from a wide range of backgrounds. Schools were initially contacted by email/letter, outlining the aims and objectives of the study, the practical implications of the research and what it would involve. This was then followed up by a telephone call. If a school showed an interest in being involved, a presentation was given to the students and staff by the research team. Once a school agreed to participate, a letter was sent to the parents/guardians for all students in the relevant school years (years 5–10) with information on the study for both the parents/guardians and the students plus a consent form. Students who were consented by their parents/guardians were invited to participate in the study, including those who were undergoing orthodontic treatment. The list of consented students was shared with the schools and mutually suitable dates were arranged for the research teams to visit the schools and collect the data.

The data were collected on two separate visits to the school. Initially, a team of psychologists from Goldsmiths, University of London and the University of Kent went into the school and administered the questionnaire, either via physical copies or online using Qualtrics (Qualtric XM, Provo, UT, USA), dependent on the school’s preference. Before completing the questionnaire, each consented student was asked to fill out an assent form to confirm they still wanted to participate in the study. A few days later, the consented students were invited to undergo a clinical examination by a consultant orthodontist (AD). This was undertaken individually at the school in the presence of a dental nurse with the student sitting down illuminated with a portable angle-poised light and using a dental mirror and a millimetre ruler.

Recruitment commenced in December 2017 and finished in December 2019.

### Sample size calculation

As the main aim of this exploratory study was to examine whether bullying and malocclusion are independent, we planned for chi-square analyses. At the time of planning this study, the general consensus was that that for a 2 × 2 (Bullying: Yes-No, Malocclusion: Yes-No) chi-square analysis of independence, at least 80% of the expected cell values should be at least 5 or higher. Furthermore, the percentage of school students who have reported bullying varies hugely across sources (5%–20% in Smith (2014) and 17% in DfE (2018). Moreover, bullying or teasing in children referred for orthodontic treatment in the UK has been reported at 12.8%–38% (Fleming et al., 2008; Seehra et al., 2011). However, as we wanted to be conservative with our sampling approach, and in order to also account for drop-out and non-attendance, we planned for a recruitment of 1000 participants, aiming to identify at least 50 cases of bullying based on the 5%–20% reported by Smith (2014).

### Measurement of bullying

There are two aspects to be considered with respect to bullying: the frequency with which it occurs and the type of bullying. To measure these, a shortened version of the Olweus bullying questionnaire was used (Olweus, 1996). This is a valid and internationally recognised questionnaire that assesses perpetration/victimisation related to specific forms of bullying (Green et al., 2013). The questionnaire includes a definition of bullying at the start, consisting of three factors: an intent to cause harm; repetitive in nature; and an imbalance of power between victim and perpetrator. This is followed by self-report questions that assess events related to bullying behaviours using a referential period of 2 months. Each question has five or more responses using a Likert-type scale. For questions looking at frequency of bullying, the response options are: it hasn’t happened to me in the past couple of months; only once or twice; two or three times a month; about once a week; and several times a week (scoring 0–4). Each question is scored separately. Based on the question ‘How often have you been bullied at school in the past couple of months?’, a cutoff for the definition of being bullied has previously been described as a reported frequency of two or three times a month or greater (Solberg and Olweus, 2003); this was applied to categorise participants into ‘bullied’ and ‘not bullied’.

The questionnaire also included questions on type of bullying with responses based on the frequencies as above. The following questions were used to measure the different types of bullying:

*Called names*: ‘I was called mean names, was made fun of or teased in a hurtful way.’
*Excluded*: ‘Other students left me out of things on purpose, excluded me from their group of friends or completely ignored me.’
*Physical bullying*: ‘I was hit, kicked, pushed and shoved around.’
*Mean comments*: ‘I was bullied with mean names or comments.’
*Mean comments of a sexual nature*: ‘I was bullied with mean names, comments or gestures with a sexual meaning.’
*Cyberbullying*: ‘I was bullied with mean or hurtful messages, calls or pictures, or in other ways on my mobile phone, over the Internet or on social media.’

The same cutoff of a reported frequency of two or three times a month or greater was applied to the questions related to the different types of bullying to group the participants into ‘bullied’ and ‘non-bullied’.

As the aim was to assess the relationship between malocclusion and being a victim of bullying, as opposed to an instigator, only the questions related to victimisation were included, as has been done in a previous study (Seehra et al., 2011). As the questionnaire gives item-specific scores and does not calculate an accumulative score, this did not affect the validity.

The data were anonymised, coded and entered into a spreadsheet in SPSS version 26.0 (IBM Corp., Armonk, NY, USA).

### Clinical assessment

A clinical examination was carried out by a consultant orthodontist (AD) who was calibrated in the use of the Index of Orthodontic Treatment Need (IOTN). The features that were recorded are listed in Table 1. Teeth were considered impacted when there was less than 4 mm of space for them to erupt, or in the case of the maxillary canines, they remained unerupted beyond the normal eruptive age and there was no mobility of the primary teeth. Similarly, teeth were considered missing if they were not present beyond the normal eruptive age and the primary tooth was not mobile. While ideally a radiograph would have been taken to confirm this, this was neither practical nor ethical and the IOTN can be used as a screening tool without radiographs with good validity and reliability (Brook and Shaw, 1989).

**Table 1.** Traits recorded during clinical examination.

Feature	How assessed	Classification
Skeletal relationship	Extra-orally in profile with head in natural head posture	I / II / III
Lips	Extra-orally from the front	Competent / incompetent
Incisor show at rest	Extra-orally from the front	Measurement in millimetres
Incisor classification	Intra-orally	I / II div 1 / II div 2 / III
Overjet	Intra-orally	Measurement in millimetres
Overbite	Intra-orally	Average / increased / reduced / anterior open bite
Crowding/spacing	Intra-orally	Well aligned / mild / moderate / severe / spaced
Impaction of teeth	Intra-orally	Yes / no
Index of Treatment Need (IOTN)	Intra-orally	Dental Health component 1–5 Aesthetic component 1–10
Current orthodontic status	Intra-orally	None / fixed appliance / removable appliance / functional appliance / retention

The data were recorded on standardised data collection sheets, which were anonymised and coded. Information on ethnicity was collected using the global categories used in the 2011 census (Office for National Statistics et al., 2017). The data were then entered into a spreadsheet in SPSS version 26.0 (IBM Corp.).

### Reproducibility

Approximately 10% of the sample underwent a second clinical examination on a separate occasion by the same examiner (AD). Intra-examiner reliability was measured using weighted kappa for the numeric ordinal IOTN Dental Health (DHC) and Aesthetic component (AC) repeated scores.

### Data analysis

A statistical analysis was performed using SPSS version 26.0 (IBM Corp.). To assess the relationship between the presence of one or more traits of malocclusion and self-reported bullying, the bullying data were dichotomised based on the self-reported frequency. A participant was classified as bullied if they reported being bullied two or three times a month in the last 2 months or greater (Solberg and Olweus, 2003). As this was an initial exploratory analysis looking at whether the data matched the expected frequencies, contingency tables were created according to whether or not students were bullied. Chi-square tests were used to investigate whether bullying and other nominal and ordinal data were independent. Where the expected cell frequencies were less than 5, Fisher’s exact test was used.

## Results

Of the 53 schools approached, 16 agreed to take part. The schools consisted of eight primary schools and eight secondary schools. All the primary schools were mixed state schools. Two of the secondary schools were selective grammar schools, one mixed and one ‘single-sex school’ (girls only). ‘Single sex’ is the term used by this school’s Local Education Authority to refer to schools that historically educate either male or female pupils. The other six schools were non-selective, five being mixed and one being defined by their local authority as a ‘single-sex school’ (girls only, see above definition).

Due to the COVID-19 pandemic, the data collection was concluded before we managed to recruit 1000 participants. Out of a potential recruitment pool of 3750 students across the 16 schools, 948 students were consented to take part, representing a 25% response rate. Of these, 768 students completed the bullying questionnaire and 755 subsequently underwent the clinical examination with 698 completing both the questionnaire and undergoing the orthodontic examination. A subsequent post-hoc power analysis revealed that the estimate of 5% bullying cases used for the sample size calculation was too conservative and the 9.7% bullying cases observed in this study yielded an observer power of 98%, which was therefore perceived to be adequate for the study. The post-hoc power analysis was performed using G*power 3.1.9.6. (Faul et al., 2007).

## Reproducibility

The weighted kappa values were 0.962 for IOTN DHC (almost perfect agreement) and 0.733 for IOTN AC (substantial agreement) (Landis and Koch, 1977).

## Characteristics of the non-bullied and bullied participants

Using the above criteria, 68 participants were classified as being bullied. Table 2 shows the sociodemographic characteristics of the self-reported bullied and non-bullied groups. Using chi-square tests of independence, this showed no relationship between the two groups in prevalence of bullying in relation to age, gender and ethnicity (all *P* >0.14). In terms of types of schools, there was a relationship between the prevalence of bullying and school type, with a significantly higher prevalence of bullying reported in mixed-sex schools as opposed to single-sex schools ((1, *N* *=* 698) = 4.97, *P* = 0.03). There was also a higher prevalence reported in primary schools as opposed to secondary schools and non-selective schools as opposed to selective schools, although this did not reach statistical significance (*P* = 0.05 and 0.06, respectively).

**Table 2.** Comparison of sociodemographic characteristics of bullied and non-bullied participants.

Variable	Total in group	Bullied	*P* value
*Age (years)*			
10	94	14 (14.9)	
11	118	11 (9.3)	
12	241	26 (10.8)	
13	150	8 (5.3)	
14	75	6 (8.0)	
15	14	2 (14.3)	0.17
*Gender*			
Male	275	32 (11.6)	
Female	421	36 (8.6)	
Other	2	0 (0)	0.34
*Ethnicity*			
White	490	53 (10.8)	
Asian	63	1 (1.6)	
Black / African / Carribean	46	5 (10.9)	
Mixed / multiple	55	7 (12.7)	
Other ethnic group	18	1 (5.6)	
Rather not say	26	1 (3.8)	0.14
*Primary vs. secondary schools*			
Primary	169	23 (13.6)	
Secondary	529	45 (8.5)	0.05
*Mixed vs. single-sex schools*			
Single sex (girls)	157	8 (5.1)	
Mixed	541	60 (11.1)	0.03
*Selective vs. non-selective schools*			
Selective	144	8 (5.6)	
Non-selective	554	60 (10.8)	0.06

Values are given as n (%).

## Malocclusion and bullying

### Overjet and bullying

**Figure 1.**
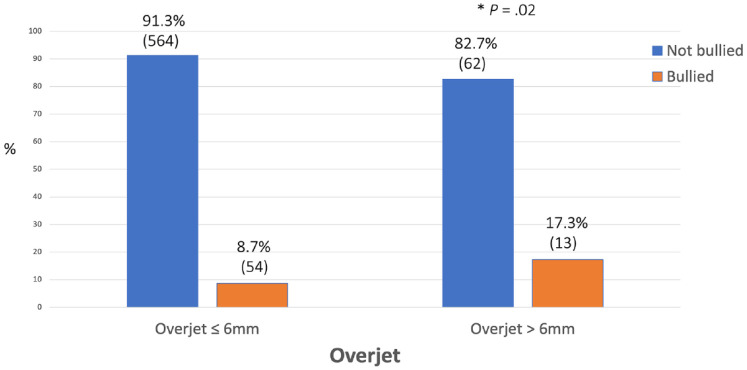
Percentage of reported bullying for overjet.

A chi-square test of independence between frequency of bullying and overjet revealed a significant relationship between the two variables for an increased overjet of greater than 6 mm ((1, *N* *=* 693) = 5.66, *P* = 0.02) (Figure 1).

### IOTN DHC and bullying

**Figure 2.**
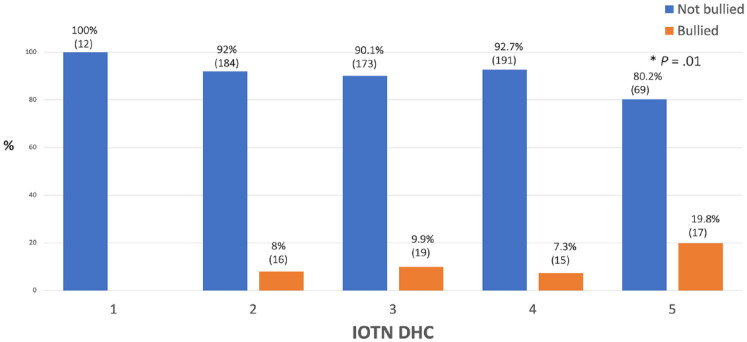
Percentage of reported bullying for IOTN DHC.

A chi-square test of independence between frequency of bullying and IOTN DHC, revealed a significant relation between the two variables ((4, *N* *=* 696) = 13.4, *P* = 0.01). Looking at the standardised residuals, for the groups of 1, 2, 3 and 4 IOTN DHC, the prevalence of bullying was independent from the IOTN DHC scores. However, the two variables, IOTN DHC and prevalence of bullying, were not independent from IOTN 5 (Figure 2).

### IOTN AC and bullying

**Figure 3.**
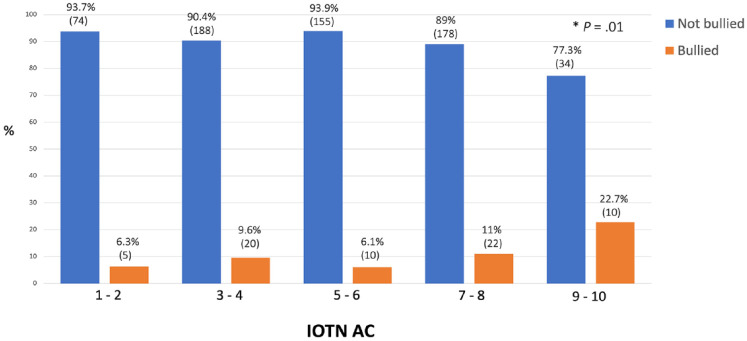
Percentage of reported bullying for IOTN AC.

Due to the small sample sizes at the extremes of the IOTN AC scores, the scores were grouped into pairs. A chi-square test of independence between frequency of bullying and IOTN AC, revealed a significant relation between the two variables ((4, *N* *=* 696) = 12.5, *P* = 0.01). Looking at the standardised residuals, for the groups 1–2, 3–4, 5–6 and 7–8 IOTN AC, the prevalence of bullying was independent from the IOTN AC scores. However, the two variables, IOTN AC and frequency of bullying, were not independent from group IOTN AC 9–10 (Figure 3).

### Other traits of malocclusion and bullying

**Figure 4.**
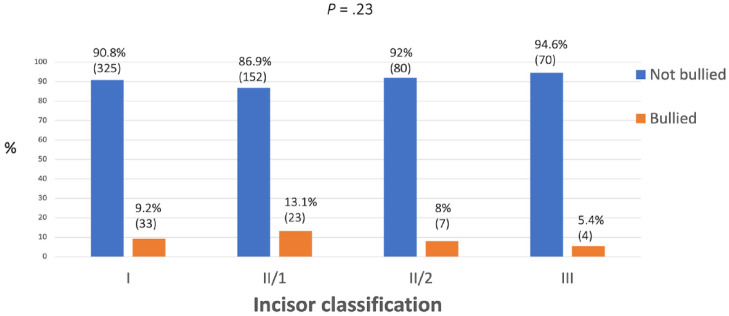
Percentage of reported bullying for incisor classification.

**Figure 5.**
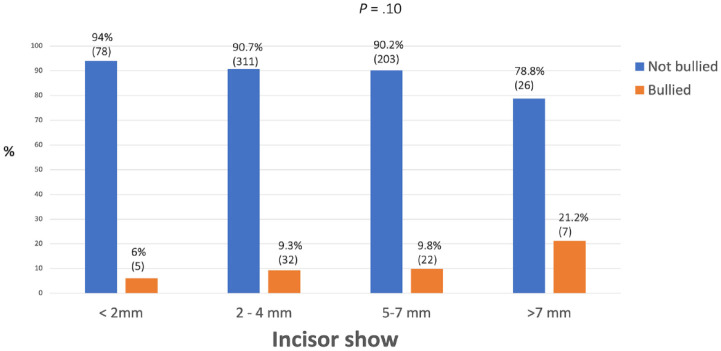
Percentage of reported bullying for maxillary incisor show at rest.

**Figure 6.**
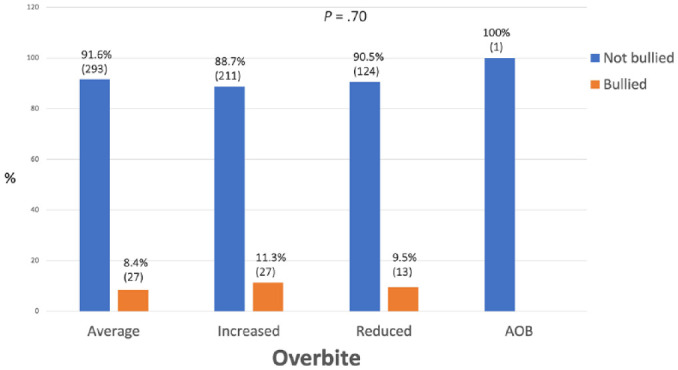
Percentage of reported bullying for overbite.

**Figure 7.**
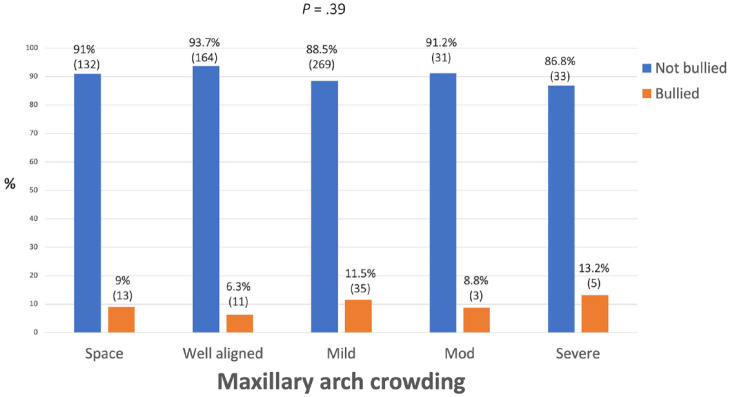
Percentage of reported bullying for maxillary crowding/spacing.

There was no relationship found between prevalence of bullying and incisor classification, incisor show, overbite and crowding/spacing in the maxillary arch, with all *P* values being ≥0.10 (Figures 4–7).

In total, 105 patients were undergoing orthodontic treatment and the prevalence of bullying reported was 12.4%, which was not statistically different from the prevalence reported in the 591 students not undergoing orthodontic treatment, which was 9.1% (*P* = 0.30). There was a statistically significant relationship in the prevalence of bullying between the 473 students who reported not or only being slightly bothered about their teeth (7.6% reported being bullied) and the 213 who reported being bothered somewhat / quite a lot / a lot bothered (14.1% reported being bullied) *(*(1, *N* *=* 686) = 7.08, *P* = 0.008).

## Type of bullying analysis

### Gender and different types of bullying

Table 3 shows the prevalence of different types of bullying in relation to gender. There was a significant relationship between gender and physical bullying, with a higher prevalence reported in boys ((2, *N* *=* 665) = 23.3, *P* <0.001). This was the same for being called names ((2, *N* *=* 688) = 7.20, *P* = 0.03). There was no difference between boys and girls for the prevalence of being excluded or having mean comments said to them, with all *P* values being ≥0.36. There was a significant relationship between mean comments being said about them of a sexual nature and gender, with a higher prevalence in boys ((1, *N* *=* 683) = 16.3, *P* <0.001). Cyberbullying was less prevalent than physical or verbal bullying, being reported by only 23 participants, with no difference for gender. Boys tended to be bullied mainly by other boys and similarly girls tended to be bullied by other girls.

**Table 3.** Prevalence of different types of bullying in relation to gender.

Variable	Total in group	Bullied	*P*
*Called names*			
Male	274	40 (14.6)	
Female	414	39 (9.4)	0.03
*Mean comments*			
Male	271	30 (11.1)	
Female	416	34 (8.2)	0.36
*Excluded*			
Male	272	16 (9.6)	
Female	417	47 (11.3)	0.62
*Physical violence*			
Male	256	33 (12.9)	
Female	409	13 (3.2)	<0.001
*Mean comments of sexual nature*			
Male	271	23 (8.5)	
Female	412	8 (1.9)	<0.001
*Cyberbullying*			
Male	261	10 (3.8)	
Female	411	13 (3.2)	0.64

Values are given as n (%).

### Malocclusion and different types of bullying

Table 4 shows the prevalence of different types of bullying in relation to the traits of malocclusion found to have an overall relationship with bullying. The IOTN DHC and AC scores have been dichotomised into groupings where a significant difference was found for overall self-reported bullying (IOTN DHC 1–4 vs. 5 and IOTN AC 1–8 vs. 9–10). There was a significant relationship between an overjet of greater than 6 mm and being called names ((1, *N* *=* 685) = 8.28, *P* = 0.004), having mean comments made ((1, *N* *=* 684) = 6.32, *P* = 0.01) and being excluded ((1, *N* *=* 687) = 6.01, *P* = 0.01) but not of physical violence (*P* = 0.27). There was a significant relationship between IOTN DHC 5 and AC 9 or 10 and being called names ((1, *N* *=* 688) = 4.25, *P* = 0.04 and (1, *N* *=* 687) = 6.66, *P* = 0.01) and having mean comments made ((1, *N* *=* 687) = 8.26, *P* = 0.004 and (1, *N* *=* 686) = 4.99, *P* = 0.03) but not for being excluded and physical violence.

**Table 4.** Prevalence of different types of bullying in relation to malocclusion.

Variable	Total in group	Bullied	*P*
*Called names*			
Overjet ⩽6 mm	611	63 (10.3)	
Overjet >6 mm	74	16 (21.6)	0.004
IOTN DHC 1–4	606	64 (10.6)	
IOTN DHC 5	82	15 (18.3)	0.04
IOTN AC 1–8	645	69 (10.7)	
IOTN AC 9–10	42	10 (23.8)	0.01
*Mean comments*			
Overjet ⩽6 mm	609	51 (8.4)	
Overjet >6 mm	75	13 (17.3)	0.01
IOTN DHC 1–4	603	49 (8.1)	
IOTN DHC 5	84	15 (17.9)	0.004
IOTN AC 1–8	644	56 (8.7)	
IOTN AC 9–10	42	8 (19)	0.03
*Excluded*			
Overjet ⩽6 mm	612	58 (9.5)	
Overjet >6 mm	75	14 (18.7)	0.01
IOTN DHC 1–4	604	59 (9.8)	
IOTN DHC 5	85	14 (16.5)	0.06
IOTN AC 1–8	646	65 (10.1)	
IOTN AC 9–10	42	8 (19)	0.07
*Physical bullying*			
Overjet ⩽6 mm	595	39 (6.6)	
Overjet >6 mm	69	7 (10.1)	0.27
IOTN DHC 1–4	582	37 (6.4)	
IOTN DHC 5	83	9 (10.8)	0.13
IOTN AC 1–8	625	43 (6.9)	
IOTN AC 9–10	39	3 (7.7)	0.85

Values are given as n (%).

## Discussion

This cross-sectional mixed-methods study of reported bullying in schoolchildren aged 10–14 years in the South East of the UK found a self-reported prevalence of 9.7%. An increased overjet and great or high need for orthodontic treatment, as measured by IOTN DHC and AC, were significantly associated with being bullied. The bullying related to malocclusion was primarily verbal, being called rude names and having mean comments made, rather than physical or being excluded. Being bullied was also significantly associated with being in a mixed-sex as opposed to a single-sex all-girls school. Caution should be exercised when interpreting the results, particularly in relation to the subgroup testing, as some of the subgroups were very small.

## Limitations

A limitation of this study is the level of missing data resulting in part from the survey and dental examination data being collected over two separate data collection points, on different days. This was for logistical and ethical reasons and to try and reduce selection and measurement bias, with the questionnaire being completed on a separate occasion from the clinical examination, with no prior knowledge of the results of either. However, this meant that there was a group of consented participants who either completed the questionnaire or the clinical examination, but not both. This resulted in a high number of incomplete datasets and missing data. However, this drop-out was accounted for in the sample size calculation and was random.

There was a larger number of girls than boys recruited into this study: this was coincidental being due to the schools that consented to take part. However, the frequency of bullying reported was very similar between the genders. A series of large surveys of 10–15-year-olds in the UK carried out between 2013 and 2018 found a larger number of girls reporting being bullied (DfE, 2018). This was not found in this study, which found a reduced prevalence of bullying reported in both the single-sex all-girls and selective schools.

The prevalence of bullying reported in this study is lower than previously reported in the UK for this age group. This may have been due to underreporting of bullying. An opt-in consent process was employed, meaning individuals who were experiencing victimisation may have been more reluctant to be involved for fear of becoming a target for further bullying. The study also involved a dental examination, which may have discouraged participation by more anxious individuals. As high levels of anxiety are associated with being bullied, this may in part explain the lower prevalence of bullying recorded. The lower prevalence reported could also be due to the strict definition of bullying in the Olweus questionnaire used, that of being bullied two or three times a month in the last 2 months or greater (Solberg and Olweus, 2003). The 17% reported by the Office for National Statistics survey in the UK was for 10–15-year-olds who reported being bullied in the last year (DfE, 2018). Looking at the frequency of bullying reported in this survey, of the 17% who reported being bullied in the last year, 32% said they were bullied at least once a week over the last year and 12% said they were bullied once every 2–4 weeks, the latter corresponding to the frequency used as a definition of bullying in this study. The remaining participants reported that the frequency varied, they were bullied less often or they did not know/want to answer. This suggests that if the criteria used in this study were applied in the DfE study, the overall prevalence of bullying would probably have been lower than 17%.

While the overall sample and the number of students who reported being bullied gave the study sufficient power for the analysis in relation to malocclusion and bullying, it must be noted that some of the subgroups tested were very small, meaning that the results need to be interpreted with caution. The only way to overcome this would have been to recruit a much larger sample group, which was made impossible by the restrictions imposed by the COVID-19 pandemic. However, we still feel it is important to report these results as it gives a greater insight into the frequencies and types of bullying being experienced by students with a malocclusion.

## Interpretation and comparison with other studies

The relationship between increased overjet and bullying has been reported in a sample of 10–14-year-olds referred for orthodontic treatment in the UK (Seehra et al., 2011). That study also found a higher prevalence of bullying in individuals with a high IOTN AC but not DHC. However, this was based on a sample referred to a hospital orthodontic department and, as such, would be skewed to include a higher percentage of more severe malocclusions. It was therefore unlikely to have a normal distribution for malocclusion. The present study, in contrast, investigated a sample of school children and should therefore be more representative of the population in the schools sampled and in state education in the UK. Indeed, the prevalence of the traits of malocclusion reported in this study is similar to previous findings for European population groups (Lombardo et al., 2020). In the present study, 15% of the sample were undergoing orthodontic treatment. This again is very similar to levels reported nationally in the UK, with 9% of 12-year-olds and 18% of 15-year-olds undergoing orthodontic treatment (Rolland et al., 2016). Unmet treatment need, as measured by IOTN DHC with a score of 4 or 5, was 42%. This was higher than previously reported in the UK, with 37% for 12-year-olds and 20% for 15-year-olds (Rolland et al., 2016). This may be because this study included children aged 10 and 11 years, many of whom were yet to start orthodontic treatment. It also may be reflective of the opt-in nature of the consent, which may well have encouraged more children who had a malocclusion and a desire for treatment to participate while those with no malocclusion or milder malocclusions had less interest to be involved. Overall, however, as the figures are very similar, we feel the sample group used in the present study was representative of the population in the schools sampled and more broadly schools in the UK.

The results of this study were different to another large cross-sectional study carried out in the UK that did not find a relationship between bullying and increased overjet (Agel et al., 2014). While that study used the same cutoff of greater than 6 mm to define an increased overjet, the prevalence reported was much lower (1.5% as opposed to 11%). This maybe because that study looked at 15- and 16-year-olds, a time at which most orthodontic treatment would be completed and an increased overjet corrected. Combined with the reduction in prevalence of bullying with age, this may explain why a relationship was not found (DfE, 2018).

Seehra et al. (2011) found higher levels of bullying reported in patients with deep overbites. This relationship was not found in the present study. This is difficult to explain and may represent a type 1 error due to the high correlation between an increased overbite and an increased overjet in the sample group used by Seehra et al. (2011), who were children and adolescents referred for treatment.

Other studies have reported higher levels of bullying in individuals with anterior spacing (Al-Bitar et al., 2013) or crowding in the maxillary arch (Duarte-Rodrigues at al., 2020; Rivera-Montoya et al., 2020). This was not found in this study. This may be because the clinical examination used in the present study recorded spacing and crowding in both dental arches but not its location. There are also some key methodological differences between the current research and these previous studies: Al-Bitar et al. (2013) relied on a self-reported questionnaire and did not involve a clinical examination.

Two systematic reviews have concluded that there was very low-quality evidence that a conspicuous extreme malocclusion may be related to the occurrence of bullying among children and adolescents, which is supported by this study (Broutin et al., 2023: Tristao et al., 2020). All of the studies included in these systematic reviews were cross-sectional in nature, looking at either a sample of schoolchildren or a sample of children and adolescents referred for orthodontic treatment. Bullying was measured using a variety of questionnaires. In this study, the Olweus bullying questionnaire was used. This is one of the most used and validated questionnaires on bullying in schoolchildren, having been translated into several languages and used in numerous studies internationally. It also has the advantage that it includes a definition of bullying at its beginning, a specific reference time frame for the events to have occurred and a cutoff to identify someone who would be defined as being bullied, and frequency items, which provide a more exacting measure of bullying instance (Solberg and Olweus, 2003). This builds on previous studies that often use questionnaires related to bullying or teasing with simple yes or no response options, and with no definition or time frame provided. This difference may explain the higher incidence of bullying reported in other research, compared to the present study, as in the present study participants were reporting within a strict definition and time frame.

As this is a cross-sectional study, the longitudinal and directional relationship between experience of bullying, malocclusion and how young people feel about their teeth (how bothered they are by them) remains to be tested. Furthermore, as it is an initial exploratory analysis, it does not include multivariate analyses controlling for confounding factors and therefore can only make inferences based on associations. Indeed, when confounding variables are accounted for, a recent systematic review found no association between malocclusion and bullying (Miranda e Paulo et al., 2022). Further analysis is planned on the dataset used in this study using a multivariate analysis.

## Generalisability

The sample included in this study was taken from a broad range of state schools in the South East of the UK. Despite the slightly lower level of bullying reported compared to national survey data, the sample presented with similar levels of malocclusion and orthodontic treatment need previously reported in the UK (Rolland et al., 2016). As such we feel it is fairly representative of the larger population and the findings can be generalised to schoolchildren aged 10–14 years in the UK.

Therefore, despite the limitations of this study, we feel this study builds on the growing body of research that shows there is a relationship between an aesthetically handicapping malocclusion and an increased risk of being bullied in childhood and adolescence. Considering the negative impact bullying can have in both the short and long term, it is important that parents, schools and dentists are made aware of this relationship and schoolchildren with a malocclusion who are being bullied are referred in an appropriate and timely manner for possible treatment. What limited evidence we have does show that orthodontic treatment can be very beneficial in this group of patients (Seehra et al., 2013). It is, therefore, important adequate resources continue to be allocated to allow treatment of these extremely deserving group of patients.

## Conclusion

In this cross-sectional study involving schoolchildren aged 10–14 years in the UK, a prevalence of bullying of 9.7% was found with no significance difference for age, gender or ethnicity. There were lower levels of bullying reported in single-sex, girls’ schools and selective schools. Boys were at greater risk of physical bullying and being called names. The prevalence of malocclusion and need for orthodontic treatment as classified using IOTN DHC 4 and 5 was 42%. Having an increased overjet and a more severe malocclusion / greater need for orthodontic treatment as measured by IOTN DHC and AC was associated with being bullied.
